# Formation of a Complex Topologies of SAW-Based Inertial Sensors by Laser Thin Film Local Evaporation

**DOI:** 10.3390/mi12010010

**Published:** 2020-12-24

**Authors:** Alexander Kukaev, Dmitry Lukyanov, Denis Mikhailenko, Daniil Safronov, Sergey Shevchenko, Vladimir Venediktov, Andrey Vlasov

**Affiliations:** 1 Department of Laser Measurement and Navigation Systems, St. Petersburg State Electrotechnical University, 197022 St. Petersburg, Russia; askukaev@gmail.com (A.K.); dplukyanov@mail.ru (D.L.); kratosloaded@mail.ru (D.M.); daniilsafronov@gmail.com (D.S.); syshevchenko@mail.ru (S.S.); 2 Department of Physics, St. Petersburg State University, 197022 St. Petersburg, Russia; 3 Institute of Chemistry, St. Petersburg State University, 197022 St. Petersburg, Russia; drew-v@yandex.ru

**Keywords:** solid-state microgyroscope, surface acoustic wave, laser, topology, delay line

## Abstract

Originally, sensors based on surface acoustic waves are fabricated using photolithography, which becomes extremely expensive when a small series or even single elements are needed for the research. A laser thin film local evaporation technique is proposed to substitute the photolithography process in the production of surface acoustic wave based inertial sensors prototypes. To estimate its potential a prototype of a surface acoustic wave gyroscope sensing element was fabricated and tested. Its was shown that the frequency mismatch is no more than 1%, but dispersion of the wave on small inertial masses leads to a spurious parasitic signal on receiving electrodes. Possible ways of its neglecting is discussed.

## 1. Introduction

The application fields of microelectromechanical sensors (MEMS) are becoming wider every year. These sensors are commonly used in consumer electronics: smartphones, laptops, and various portable devices. At the same time, it is possible to single out such areas of application (medicine, robotics and defense industry), where more stringent requirements for vibration and shock resistance are imposed on these sensors [1].

Most of MEMS design concepts assume the presence of a sensitive element (SE) in the form of inertial masses fixed on elastic suspensions (torsions), which is a limiting factor in the use of these devices on highly dynamic objects. The latter are characterized by overloads of the order of 50,000 g and angular velocities of 100,000°/s. The need for navigation sensors resistant to such impacts is confirmed by regular publications, as well as by announced grants. For example, the US Defense Advanced Research Projects Agency (DARPA) in May 2015 announced a competition for the development of an inertial navigation system based on sensors that meet the following requirements [1]: shock resistance –50,000 g, vibration resistance −50 g, measurement range −100,000°/s, dimensions −1 cm^3^.

One of promising ideas in this area is the use of solid-state microgyroscopes on bulk [2] and surface acoustic waves (SAW), which are based on the molecular kinetics of a solid body. Today, such sensors are being actively developed [3,4,5,6,7,8,9,10,11,12,13,14,15]. At the same time, the technology of manufacturing a sensitive SAW gyroscope has its own difficulties, due to the need to manufacture a complex electrode topology. Traditional methods of its formation are based on the use of photolithography. This method is rather laborious and does not have universality, since it involves many operations and requires the manufacture of special photomasks for each specific topology. In this work, we propose to use the method of local laser evaporation as an alternative method for creating electrode topologies. Its essence lies in the fact that with the help of laser radiation, which is positioned on a plane, it is possible to create drawings of any complexity on pre-metallized piezomaterial plates. This approach is versatile and does not require the design and manufacture of photomasks when changing the topology geometry. The purpose of this work is to manufacture a prototype of the sensitive element of a SAW-based solid-state microgyroscope using the indicated method and to check its quality, i.g., the frequency matching, insertion loss value, parameters of possible parasitic signals.

## 2. The Scheme of Construction of a Solid-State Microgyroscope on SAW

**Figure 1:**
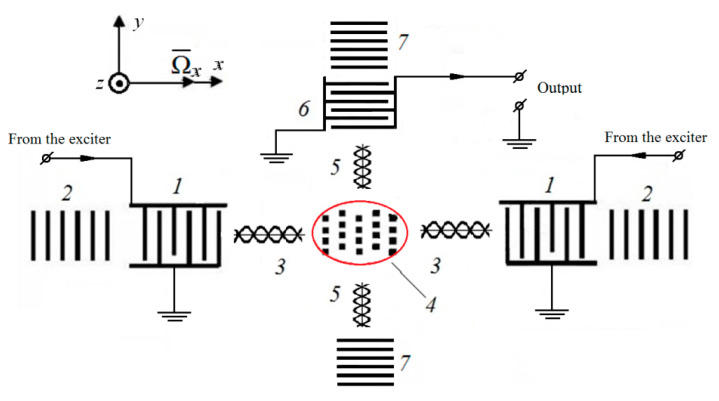
Scheme of the sensitive element of a SAW-based solid-state microgyroscope with cross-links and a matrix of inertial masses.

Let us consider an example of the construction of a SE of a solid-state microgyroscope on standing SAWs [4] (Figure 1).

According to Figure 1, with the help of exciting interdigitated transducers (IDTs) 1 and reflectors 2, a primary standing wave 3 is generated, in the antinodes of which miniature masses are placed, forming a matrix 4. Together with the particles of the acoustic duct, they vibrate along the *z* axis. In the presence of angular velocity ${\bar{\Omega }}_{x}$, the Coriolis acceleration and the corresponding forces *F*_C_ = *mV*_SAW_ orthogonal to the direction of the primary wave 3 arise. Under its action, the particles of the acoustic duct, together with the attached masses, perform oscillatory movements along the *y* direction, arousing a secondary SAW 5, which is sensed by the IDT 6.

The choice of the primary wave frequency is dictated by the sensitivity of the solid-state microgyroscope. An increase in sensitivity associated with a decrease in frequency was predicted by Lao in 1980 [16]. In his work, the author claims that with a decrease in the natural frequency of the surface acoustic wave, the ratio of the increment in the phase velocity of the SAW to its speed itself increases, which is demonstrated by the following expression:

(1) $$\frac{\Delta V}{V_{0}}=\beta \frac{\Omega }{\omega },$$

where

(2) $$\beta =\frac{\xi }{{2(1-\xi }^{2})}\frac{{\left(1-\eta^{2}\xi^{2}\right)}^{1/2}(\eta^{2}\xi^{-2}-4)-{\left(1-\eta^{2}\right)}^{1/2}(\eta^{2}-4)}{(\eta^{2}-2)+(1+\xi^{2}-2\xi^{2}\eta^{2}){\left(1-\xi^{2}\eta^{2}\right)}^{-1/2}{\left(1-\eta^{2}\right)}^{-1/2}}$$

(3) $$\xi =\frac{V_{T}}{V_{L}},\;\eta =\frac{V_{0}}{V_{T}},$$

where *V*_0_—phase velocity of the SAW at Ω = 0; *V*_L_—longitudinal wave phase velocity; *V*_T_—shear wave phase velocity; Δ*V*—phase velocity increment; Ω—angular velocity; ω—SAW carrier frequency. Thus, it was analytically shown that the transition to the low-frequency regime makes it possible to increase the scale factor of a solid-state microgyroscope based on SAW, while not loading the topology with additional elements in the form of a matrix of inertial masses.

The practical implementation of the described scheme by the photolithography method turned out to be economically inexpedient, since the production of special photomasks for one prototype according to the calculated data was about half a year. Changing the geometry of the pattern on the surface of the sample would require a repeated procedure for making special masks.

**Figure 2:**
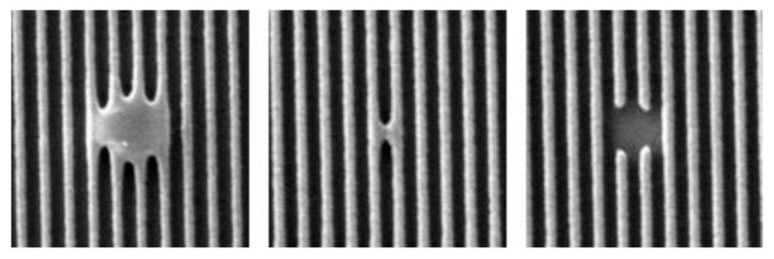
Examples of photolithography faults in IDT structure.

Also traditional production method has a chance of geometry faults (Figure 2) that may affect the generation frequency or even short-circuit the transducer.

A promising direction in the manufacture of a SE of a SAW-based solid-state gyroscope is the use of modern flexible technological processes that will make it possible to make changes to the current production process without significant time and financial costs. These advantages are possessed by the method of local laser evaporation [17]. Let us consider in more detail the principle of its operation.

## 3. Local Laser Evaporation Method

**Figure 3:**
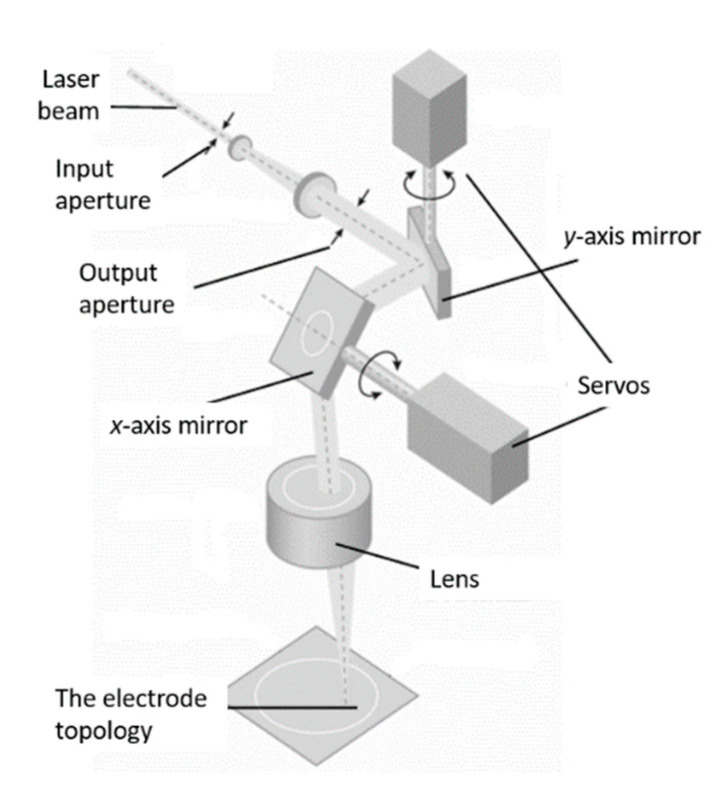
Structural scheme of the setup for local laser evaporation.

The essence of this technology is reduced to the removal of a part of the film coating, originally deposited on the substrate, using evaporation caused by laser radiation. This technique is often referred to as laser ablation. First, a film layer, which is usually represented by aluminum with a thin vanadium sublayer, is applied to the surface of the substrate. Next, a laser, which is turned to a pulsed mode for greater efficiency of the process, draws the desired electrode topology on the film. Several strict requirements are imposed on this process regarding the radiation power, reproducibility of the size and shape of the light spot, as well as the stability of the entire system. This method of processing products is implemented using technological laser installations. They often have a common structural scheme, regardless of the purpose and type of lasers used and contain the following main units: a source of powerful optical radiation—a laser, an optical system for directing radiation, a device for fixing a processed object, a laser control system. Figure 3 shows an example of a general structural scheme of laser installations with a scanning system.

In the method under consideration, a parallel laser beam passes through the aperture and hits a mirror, which is attached to a servo, which allows it to deflect the beam at a certain angle, for example, along the *y*-axis. After being reflected, the radiation falls on the second mirror, which deflects the laser beam along the orthogonal *x*-axis. Further, due to the lens of a flat field, the parallel beam is focused on the sample. Due to the deflection of the beam along two orthogonal axes and sequential switching on/off of the radiation, a topology pattern is formed in the plane of the acoustic duct on the deposited metal film. Accordingly, passing through the flat-field lens, the beam is focused on the surface of the film layer and an image is formed on the substrate. Depending on the size of the substrate, as well as on the type of task, the required lens is selected. Obviously, the minimum distance between two adjacent elements of the topology will be determined by the width of the laser beam in focus.

The method of local laser evaporation of metal films from an acoustic conductor allows creating complex topologies, bypassing a few technological operations used in photolithography. At the same time, there is no need to create photomasks for each range of electronic components. It is enough to download the required image from your computer. In this case, there are no consumables in the production process (photomasks must be regularly updated). The method of laser formation of topology is also a powerful tool for controlling the parameters of inertial mass matrix, since it allows configuring them in three dimensions, which is absent in modern lithographic methods. Additionally, the method provides an opportunity of topology correction after the main production process or even after the sealing in case of full-quartz packaging [18]. One more option is creation of identical topologies on both sides of the crystal wafer [17].

The speed of proposed method depends on the metallization thickness and the overall size of the desired topology. For a typical SAW-based inertial sensor SE (50 nm metal thickness, 1 cm^2^) the total procedure (except the metallization process) takes about 10 s.

**Figure 4:**
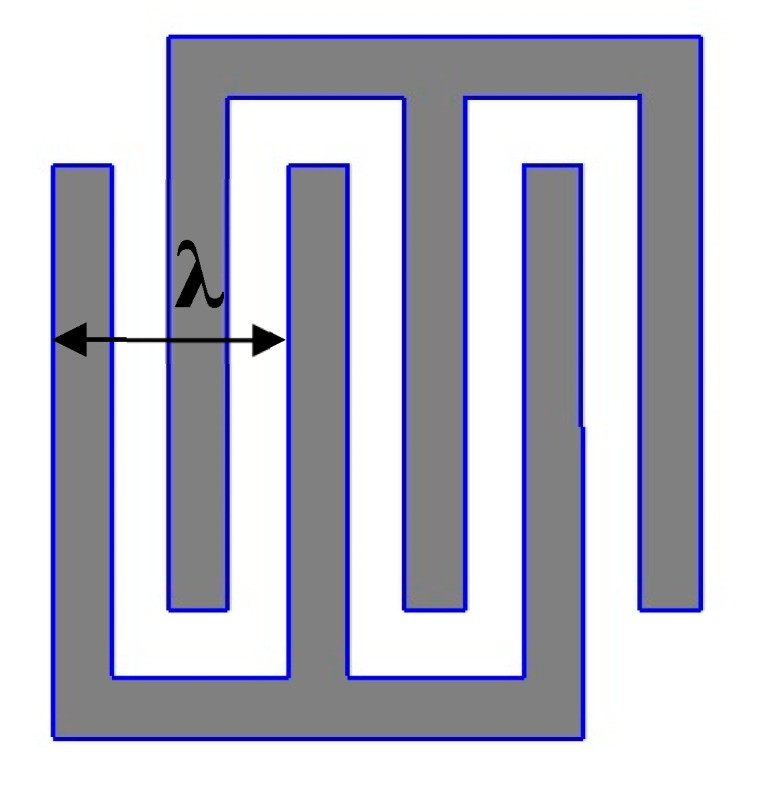
Inter-digital transducer.

The main disadvantage of the method is a significantly narrower frequency range of the manufactured sensitive elements. The upper frequency limit is determined by the smallest possible width of the laser spot in focus. Figure 4 shows the topology of the simplest IDT—the main fragment of the SAW-based SE topology.

According to Figure 3, an acoustic wavelength *λ* equal to the distance between the two nearest in-mode fingers of the IDT. Therefore, the width of the finger and the distance between them is *λ*/4. This value cannot be made smaller than the width of the laser trace left on the deposition. The frequency of the final sample is determined by the formula (4):

(4) $$f=\frac{v}{\lambda }$$

where *f*, *v*, *λ*—frequency, propagation velocity and length of SAW, respectively. Considering modern flat field focusing systems, the maximum possible value of the SAW resonator frequency (for Lithium Niobate) is 50 MHz. For comparison, the photolithography method makes it possible to produce SE with frequencies up to 1 GHz. However, as shown above, lowering the frequency of the SE of a solid-state microgyroscope increases the sensitivity of the measuring device.

## 4. Manufacturing of a SAW-Based Solid-State Microgyroscope SE Prototype Utilizing the Method of Local Laser Evaporation

To carry out experimental studies on the creation of complex topologies on the surface of SE of a SAW-based solid-state microgyroscope, a 128° YX-cut Lithium Niobate plate was used, having the shape of a circle with a diameter of 75 mm and a thickness of 1 mm. A copper coating with a thickness of 500 nm was applied to it. The choice of this material was dictated by the least distortions of the topology elements geometry caused by thermal processes occurring during laser heating [19]. Additionally, it was shown in [20] that the material should have the higher value of its density divided by its Young’s modulus.

The creation of the surface topology was carried out by local laser evaporation using a precision laser marking system “MiniMarker 2”, which main characteristics are presented in the Table 1 [21].

The width of one finger of the SE topology as well as the distance between the fingers was chosen equal to 60 μm, which corresponds to the length of the SAW *λ* = 240 μm. This value was determined as follows: on one hand, there is a width of the laser beam at the focus of 20 μm. Accordingly, it is impossible to make the distance between the electrodes smaller. Since the manufactured sample is experimental, it was decided not to produce the sensitive element at the accuracy limit of the method. On the other hand, it is necessary to limit the acoustic wavelength. Since the SAW penetrates 3*λ* deep into the substrate, and the substrate thickness is 1 mm, the maximum wavelength is 330 μm. As a result, the SAW frequency was determined by formula (4) and amounted to 16.49 MHz.

Experience of working with SAW frequency elements has shown that in the case of resonators, it is enough to have at least 100 reflectors on each side for high-quality generation of a standing wave. It is also necessary to place a 100 × 100 matrix with inertial masses in the center, each of which has dimensions *λ*/4 × *λ*/4. Since the entire topology should occupy an area of no more than a square inscribed in the circle of the substrate and considering the above structural elements, only 6 pairs were used for the input IDTs. Despite the relatively small number, it is enough to pump energy into the resulting resonator. In this case, the aperture was chosen equal to 15 mm.

The next stage of research was associated with the calculation of the operating modes of laser processing to create the highest quality topology of the pattern. To do this, it was necessary to calculate the modes of laser radiation, which would make it possible to completely evaporate the metal layer without damaging the piezoelectric substrate. As shown in [22], the shorter the duration of the laser pulse, the smaller the amount of heat removed. The maximum possible pulse duration providing the minimum heat dissipation is determined by the following relationship [19]:

(5) $$\tau \leq \frac{\pi }{4}{\left(\frac{\rho_{1}C_{1}}{\rho_{2}C_{2}}\right)}^{2}\frac{h^{2}}{a^{2}},$$

where *a*—thermal diffusivity; *C*—heat capacity; ρ—density; *h*—layer thickness; index “1” refers to the film, “2” to the substrate.

According to formula (5), for a deposited copper layer with a thickness of 500 nm deposited on a Lithium Niobate substrate, the value of *τ* will be 4 ns. Solving the system of equations for the power density, we calculate the required average laser power taking into account the pulse duration [22]:

(6) $$\{\begin{array}{c} q=\frac{T\rho Ch}{A\tau }\\ q=\frac{P}{S} \end{array}=>P=\frac{T\rho ChS}{A\tau }=2.4\;W$$

where *A*_1_—film absorption capacity; *τ*—pulse duration; *P*—average laser power; *S*—area of the beam in focus; *q*_0_—the power density of the incident radiation.

**Figure 5:**
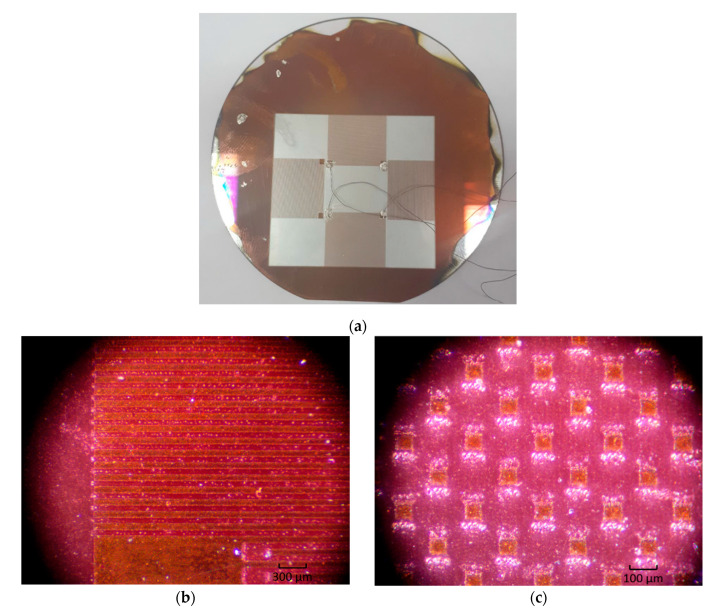
Prototype of the sensitive element of a SAW-based solid-state gyroscope under a microscope: (**a**) general view; (**b**) IDT and reflectors; (**c**) inertial mass matrix.

Calculated laser machine modes were used to create the topology of the SE of a SAW-based solid-state microgyroscope. The resulting prototype and its topology is shown in Figure 5.

Figure 5a shows a section of the resonator where the reflectors are located, as well as a part of the IDT. Measurements showed equal widths of various elements selected in arbitrary areas of the image. At the same time, attention is drawn to the absence of defects associated with metal spreading. Thus, the opposing fingers are not interconnected, which was to be achieved. The described defects inevitably arise in the photolithography method.

**Figure 6:**
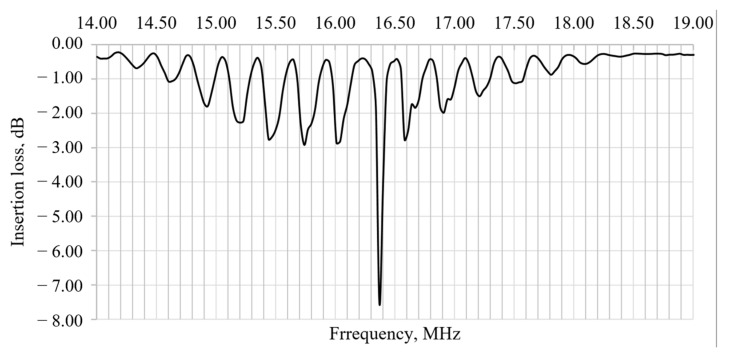
Spectrum of the primary channel of the sensitive element of a solid-state gyroscope.

Another part of the resonator is shown in Figure 5b. Shown here is the obtained matrix of inertial masses of size *λ*/4 × *λ*/4. A preliminary analysis showed that the difference in the dimensions of the elements along the *x*-axis differs from the dimensions along the *y*-axis by no more than 2–3 microns. Theoretically, this can lead to a deterioration in the propagation of an acoustic wave due to its scattering. In practice, however, these imperfections may not be significant. To assess the spectrum of the resulting prototype, electrodes were attached to the contact pads with conductive glue, which were output to the frequency connectors. Then the scheme was connected to a network analyzer, which allows spectral estimation in a wide range. The result of this work is shown in Figure 6.

As one can see from this image, the resonance peak occurs at 16.37 MHz, while the predicted frequency was 16.49 MHz. Thus, the difference between the real frequency and the calculated one is less than 1%, which indicates a high quality of performance. Also, the ratio of the central peak to the nearest harmonics is 2.68. The obtained frequency response indicates that the manufactured sensitive element can be included in the positive feedback circuit of the amplifier and, on this basis, directly form a solid-state gyroscope based on SAWs.

**Figure 7:**
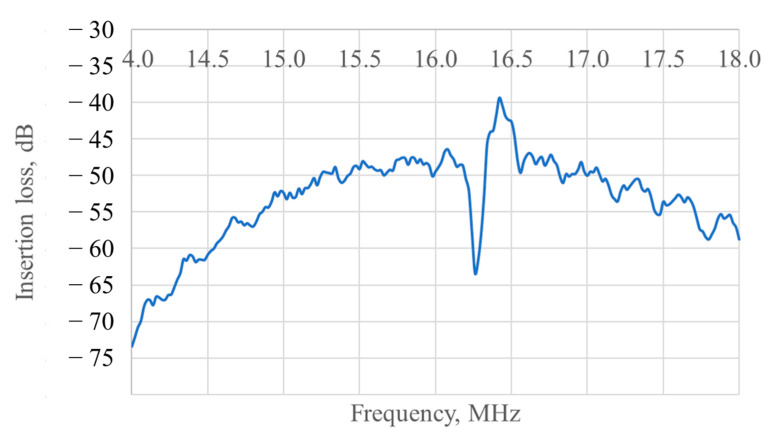
Frequency response of the prototype in the delay line regime.

Additionally the prototype was tested in the delay line regime (when one IDT in the axis becomes not an exciter, but a receiver). The frequency response for this case is shown in Figure 7.

The central frequency is the same as in the resonator regime and the insertion loss at the peak is about −40 dB which shows that the matrix of inertial masses along with the possible damage caused to the crystal surface by laser processing are not creating heavy barriers for the wave propagation.

**Figure 8:**
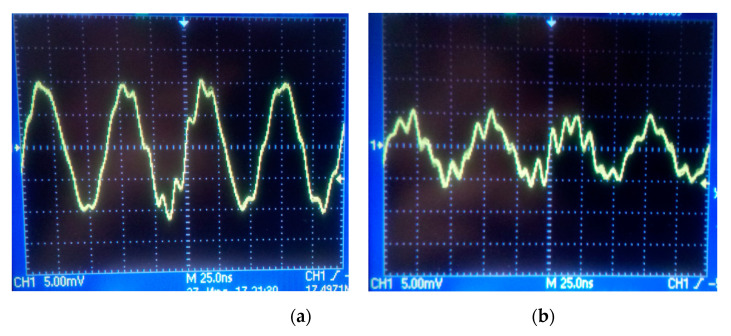
Oscillograms of the secondary channel of a SAW-based solid-state gyroscope: (**a**) spurious signal at the frequency of the SAW in the primary channel of 16.37 MHz; (**b**) spurious signal at the frequency of the SAW in the primary channel of 16.02 MHz.

However, due to the scattering from the matrix of inertial masses some part of the energy may be transferred from the primary channel to the secondary one eve in the absence of rotation. It may become a parasitic signal for the gyroscope and, therefore needs to be evaluated. For this purpose, the secondary channel was connected to an oscilloscope, and a signal of a certain frequency was fed to the interdigital transducers of the primary channel. The frequency was chosen based on the spectrum of the primary channel, and for the beginning it was taken equal to the main peak of 16.37 MHz. As a result, the following image was obtained on the oscilloscope (Figure 8a).

Figure 8a clearly shows a periodic signal with an amplitude of 10 mV_pk_, which is a relatively large value. As a result, it can be concluded that a significant part of the energy from the primary channel is transferred to the secondary one. This can lead to a significant zero shift, relative to which it will be difficult to estimate the voltage change in the presence of low angular velocities.

At the same time, attention is drawn to the signal frequency, which is 17.49 MHz, which is 1.12 MHz higher than the SAW frequency in the primary channel. This frequency divergence can be caused by several factors. First, the speed of propagation of an acoustic wave in a Lithium Niobate crystal in different directions is different. In this regard, the frequency of the orthogonal acoustic waves will also differ. Secondly, the signal arrives at the oscilloscope in a distorted state, as evidenced by the irregularities in the curve of the periodic signal in its peaks. Since the oscilloscope is sensitive to such distortions, it may not correctly display the incoming frequency. Distortions can also be introduced by parasitic electromagnetic waves, from which the sample is not shielded in any way, and due to poor contact between the wires and the electrode topology since it was organized by the conductive glue instead of a proper machine welding.

Further, during the experiment, the generation frequency in the primary channel was set to the frequency at the nearest side harmonic, which was 16.02 MHz. At the same time, the SAW amplitude in the primary channel decreased 2.2 times. The oscilloscope data are shown in Figure 8b.

It is easy to see that the amplitude of the secondary acoustic wave also decreased by 2 times and now it is 5 mV_pk_, instead of 10 mV_pk_ in the first case. Such a correlation of the two signals indicates the presence of scattering effects on the inertial mass matrix. To reduce the parasitic signal, it is important to fabricate the sides of the inertial mass with minimal distortion (less than 1% of the size of the side of the inertial mass). Also it seems promising to test round inertial masses instead of square ones.

Despite these shortcomings, in the future it is planned to develop an electrical circuit of a SAW-based solid-state gyroscope using a manufactured sensitive element. This will allow the sample to be calibrated on a rotating stand and to determine the accuracy characteristics of the gyroscope.

## 5. Conclusions

Using the proposed technique of local laser evaporation a sensing element of a SAW-based gyroscope was fabricated and further tested. The obtained sample of the SAW resonator demonstrates its performance with parameters that differ from the calculated ones by no more than 1%.

With an increase in the amplitude of the primary wave, the amplitude of the secondary one increases, which arises due to the scattering of the first on the matrix of inertial masses. In this case, at the resonant peak of the primary wave, the amplitude of the secondary signal was 10 mV_pk_. This zero shift is quite significant. It is necessary to develop an electrical circuit and test a prototype of a SAW-based solid-state gyroscope on a rotating stand. However, a preliminary conclusion can be made about the operability of the SAW resonator with cross-links and the prospects of using the method of local laser evaporation for the manufacture of SAW-based inertial sensors.

## Figures and Tables

**Table 1:** Characteristics of the high-precision laser marking system «MiniMarker 2».

Characteristic	Value
Average laser output power	20 W
Laser wavelength	1064 μm
Laser frequency	20 kHz (20,000 pulses/s)
Minimum line thickness	20 μm
Beam movement speed	Regulated
Hardware and software resolution	2.5 μm (depending on the lens)
